# Cirurgia do manguito rotador e inteligência artificial: Uma análise comparativa de qualidade e legibilidade entre ChatGPT e Gemini

**DOI:** 10.1055/s-0046-1820461

**Published:** 2026-06-16

**Authors:** Vinicius Borges Alencar, Matheus Mariano Teles, Diogo Fonseca da Cunha, Pedro Henrique Nunes Piropo, Paulo Sergio Milan Robazzi, Thiago Batista Faleiro

**Affiliations:** 1 Faculdade de Medicina da Bahia, Universidade Federal da Bahia, Salvador, BA, Brasil; 2 Universidade Federal do Triângulo Mineiro, Uberaba, MG, Brasil; 3 Hospital São Rafael, Salvador, BA, Brasil; 4 Hospital Santo Antônio, Obras Sociais Irmã Dulce (OSID), Salvador, BA, Brasil

**Keywords:** ChatGPT, educação em saúde, inteligência artificial, manguito rotador, modelos de linguagem, tecnologia da informação, artificial intelligence, ChatGPT, health education, information technology, language models, rotator cuff

## Abstract

**Objetivo:**

Ferramentas de inteligência artificial (IA) baseadas em linguagem natural, como ChatGPT-4.1 mini (OpenAI Group PBC) e Gemini 2.5 Flash (Alphabet Inc.), são utilizadas por pacientes como fonte de informação médica. Este estudo avaliou e comparou a qualidade e a legibilidade das respostas fornecidas por essas IAs, em português brasileiro, sobre cirurgia do manguito rotador.

**Métodos:**

Estudo transversal, descritivo e comparativo, com abordagem qualiquantitativa. Foram utilizadas 24 perguntas frequentes de pacientes, classificadas segundo Rothwell. Cada pergunta foi inserida individualmente nas plataformas dos dois modelos, sendo considerada apenas a primeira resposta. A qualidade foi avaliada por meio do instrumento DISCERN, desenvolvido pela University of Oxford e pela British Library, e dos critérios editoriais da Journal of the American Medical Association (JAMA). A legibilidade foi estimada com o programa Análise de Legibilidade Textual (ALT), validado para o português brasileiro. As análises estatísticas incluíram os testes de Wilcoxon, Friedman, análise de variância ([ *analysis of variance* , ANOVA, em inglês] para medidas repetidas) e *post hoc* de Conover com correção de Bonferroni.

**Resultados:**

O ChatGPT obteve escore médio DISCERN de 58,7 ± 4,0, e o Gemini, 56,3 ± 3,5, sem diferença significativa ( *p* = 0,174), mas com efeito máximo ( *rank-biserial correlation* [rrb, em inglês] = 1,0). Ambos os modelos apresentaram legibilidade média correspondente a 13,3 anos de escolaridade ( *p* = 1,000). Nenhuma resposta atendeu aos critérios editoriais da JAMA. Perguntas relacionadas a valores obtiveram os maiores escores de qualidade, ao passo que as perguntas sobre política foram as mais complexas em termos de leitura. A correlação entre qualidade e legibilidade foi moderada (ρ = 0,73; *p* = 0,099).

**Conclusão:**

ChatGPT–4.1 mini e Gemini 2.5 Flash ainda não oferecem informação médica, em português brasileiro, adequada quanto à confiabilidade editorial, qualidade e acessibilidade textual para o público leigo.

## Abstract (en)

**Objective:**

Artificial intelligence (AI) tools based on natural language, such as ChatGPT 4.1 mini (OpenAI Group PBC) and Gemini 2.5 Flash (Alphabet Inc.), are used by patients as sources of medical information. The current study aimed to evaluate and compare the quality and readability of responses provided by these AIs, in Brazilian Portuguese, regarding rotator cuff surgery.

**Methods:**

The present cross-sectional, descriptive, and comparative study followed qualitative and quantitative approaches. A total of 24 frequently-asked patient questions were used, classified according to Rothwell. Each question was entered individually into both platforms, and only the first response was considered. The quality assessment used the DISCERN instrument, developed by the University of Oxford and the British Library, and the Journal of the American Medical Association (JAMA) benchmark criteria. Readability was estimated using Análise de Legibilidade Textual (ALT, “Text Readibility Anallysis”, in Portuguese) software, validated for Brazilian Portuguese. The statistical analyses included the Wilcoxon and Friedman tests, repeated-measures analysis of variance (ANOVA), and the Conover post-hoc test with Bonferroni correction.

**Results:**

ChatGPT achieved a mean DISCERN score of 58.7 ± 4.0, and Gemini, 56.3 ± 3.5, with no significant difference ( *p* = 0.174), but with a maximum effect size (rank-biserial correlation [rrb] = 1.0). Both models showed a mean readability corresponding to 13.3 years of schooling ( *p* = 1.000). No response met the JAMA benchmark criteria. Value-based questions achieved the highest quality scores, whereas policy-related questions were the most complex in terms of readability. The correlation between quality and readability was moderate (ρ = 0.73; *p* = 0.099).

**Conclusion:**

ChatGPT 4.1 mini and Gemini 2.5 Flash do not yet provide adequate medical information in Brazilian Portuguese regarding editorial reliability, quality, and textual accessibility for the general public.

## Introdução

Modelos de linguagem natural baseados em inteligência artificial (IA), como o ChatGPT (OpenAI Group PBC) e o Gemini (Alphabet Inc.), vêm sendo amplamente utilizados por pacientes em buscas online sobre temas médicos, incluindo condições ortopédicas como lesões do manguito rotador. Essa acessibilidade imediata transforma o processo tradicional de educação em saúde, mas levanta preocupações sobre a qualidade, a confiabilidade editorial e a acessibilidade do conteúdo gerado por essas ferramentas. 1 2

Estudos recentes 3 4 5 demonstram que, embora os textos gerados por grandes modelos de linguagem ( *large language models* , LLMs, em inglês) sejam bem estruturados e semanticamente coerentes, frequentemente carecem de elementos fundamentais de rastreabilidade, como autoria identificável, data de atualização e referências bibliográficas válidas, o que compromete sua confiabilidade editorial. Além disso, a linguagem empregada tende a ser excessivamente complexa, o que dificulta sua compreensão por pacientes com menores níveis de escolaridade, o que contraria as diretrizes internacionais de legibilidade para materiais de educação em saúde. 6 7

Uma crítica metodológica recorrente na literatura é o uso de ferramentas de legibilidade baseadas no idioma inglês, o que limita sua aplicabilidade em contextos lusófonos. Para superar essa limitação, o programa Análise de Legibilidade Textual (ALT) foi desenvolvido e validado para o português brasileiro, e permite uma estimativa mais precisa da escolaridade mínima necessária para a compreensão textual. 8

Embora o uso de IAs generativas como fonte de informação médica tenha se expandido de forma progressiva no Brasil, ainda não há estudos sistemáticos que avaliem, em português brasileiro, a qualidade e a legibilidade das respostas fornecidas por versões gratuitas dessas ferramentas. Diante dessa lacuna, este estudo tem como objetivo comparar a qualidade informacional e a legibilidade das respostas produzidas pelos modelos ChatGPT 4.1 mini e Gemini 2.5 Flash, em português brasileiro, sobre cirurgia de reparo do manguito rotador, um tema frequente na prática ortopédica e de alto interesse para o público leigo.

## Métodos

### Desenho do estudo

Trata-se de um estudo transversal, descritivo e comparativo, com abordagem quantitativa e qualitativa, cujo objetivo foi avaliar e contrastar a qualidade e a legibilidade das informações fornecidas por dois modelos gratuitos de IA baseados em linguagem natural: ChatGPT-4.1 mini e Gemini 2.5 Flash. Este estudo não envolveu seres humanos ou animais, de modo que não foi necessário obter aprovação do Comitê de Ética em Pesquisa.

### Seleção dos modelos de IA

As versões gratuitas dessas ferramentas foram acessadas diretamente pelas plataformas oficiais: https://chat.openai.com e https://gemini.google.com . Nenhuma configuração personalizada, instrução contextual ou *plug-in* foi utilizado. Os testes foram realizados entre 1° e 10 de julho de 2025.

### Seleção e classificação das perguntas

Foram selecionadas 24 perguntas comumente formuladas por pacientes em contexto ambulatorial sobre o reparo cirúrgico do manguito rotador, baseadas na publicação de Warren et al. 3 ( Tabela 1 ). As perguntas foram traduzidas para o português brasileiro e submetidas a revisão por dois especialistas com experiência em ortopedia e comunicação em saúde, a fim de assegurar equivalência semântica e cultural. Em seguida, foram classificadas conforme o sistema proposto por Rothwell 9 em três categorias argumentativas: perguntas de fato (questões objetivas que buscam determinar se algo é verdadeiro e em que medida, e que podem ser respondidas com base em evidências objetivas), de política (relativas a decisões terapêuticas) e de valor (referentes a percepções subjetivas ou preferências), com oito questões por categoria.

**Tabela 1:** Lista completa de perguntas classificadas segundo Rothwell, 9 adaptada de Warren et al. 3

Fato	Política	Valor
**1. Um raio-X pode mostrar uma lesão do manguito rotador?**	1. O que acontece se uma lesão do manguito rotador não for tratada?	1. A cirurgia artroscópica do ombro vale a pena?
**2. Como ir ao banheiro após uma cirurgia no ombro?**	2. É possível esperar tempo demais para fazer a cirurgia do manguito rotador?	2. Por que a cirurgia do manguito rotador é tão dolorosa?
**3. Como posso saber se rompi meu manguito rotador?**	3. Uma lesão do manguito rotador pode cicatrizar sozinha?	3. Quanto tempo dura um reparo do manguito rotador?
**4. Quanto custa uma cirurgia do manguito rotador?**	4. Como posso acelerar a recuperação após o reparo do manguito rotador?	4. Por que a dor do manguito rotador piora à noite?
**5. Pode-se usar sutiã após cirurgia no ombro?**	5. A cirurgia do manguito rotador evita o surgimento de artrose?	5. A cirurgia do manguito rotador devolverá meu ombro ao que era antes?
**6. Quanto tempo após a cirurgia no ombro posso dirigir?**	6. O que devo fazer para voltar a praticar esportes após o reparo do manguito rotador?	6. Ainda devo fazer a cirurgia do manguito rotador se não puder pagar pela fisioterapia?
**7. Por quanto tempo é necessário dormir em uma poltrona após a cirurgia no ombro?**	7. O que acontece se eu não fizer fisioterapia após a cirurgia do manguito rotador?	7. Posso fazer a cirurgia do manguito rotador mesmo sem ter alguém para me ajudar em casa?
**8. Qual é o tempo médio de recuperação após uma cirurgia do manguito rotador?**	8. Quanto tempo posso esperar antes de fazer a cirurgia do manguito rotador?	8. A cirurgia do manguito rotador é uma cirurgia de grande porte?

### Geração e organização das respostas

Cada uma das 24 perguntas foi inserida individualmente nas interfaces web dos dois modelos de IA utilizando o idioma português brasileiro. Somente a primeira resposta gerada por cada modelo foi considerada para análise. As respostas foram transcritas integralmente, sem edição, reformulação ou exclusão de trechos, e armazenadas em formulário digital estruturado. Para cada entrada, registraram-se: a identificação da pergunta, a classificação segundo Rothwell, 9 o modelo de IA utilizado, o texto integral da resposta, os escores atribuídos na avaliação de qualidade e o índice de legibilidade.

### Avaliação da qualidade das respostas

A qualidade das respostas foi avaliada por dois revisores independentes, com experiência em análise crítica da literatura científica. Para essa análise, foram utilizados dois instrumentos validados: o questionário DISCERN 10 e os critérios de referência da *Journal of the American Medical Association* (JAMA). 11 12

O instrumento DISCERN foi desenvolvido em 1999, pela Biblioteca Britânica e pelo National Health Service Research and Development Programme, com o objetivo de avaliar a qualidade de informações sobre saúde disponibilizadas ao público em formato escrito. 10 Trata-se de uma ferramenta validada e amplamente utilizada na literatura científica para julgar a confiabilidade e a utilidade de textos educativos em saúde. O instrumento é composto por 16 itens distribuídos em 3 seções: 1) confiabilidade da informação (8 itens); 2) qualidade das informações relacionadas às opções de tratamento (7 itens); e 3) uma classificação global da qualidade do texto (1 item). Cada pergunta é pontuada em escala Likert de 1 (“não atende”) a 5 (“atende plenamente”), o que resultando em escore total mínimo de 16 e máximo de 80. Escores superiores a 70 são geralmente classificados como de qualidade “excelente”, e escores acima de 5,0 como de qualidade “boa”. As avaliações foram realizadas de forma independente por dois revisores cegos quanto à identidade do modelo de IA. Divergências foram resolvidas por consenso após nova leitura conjunta.

Os critérios de referência da JAMA englobam quatro domínios essenciais para a confiabilidade de informações em saúde: identificação da autoria, atribuição de fontes, declaração de conflitos de interesse e indicação da data de atualização. Cada critério foi avaliado de forma binária (presente = 1 ponto; ausente = 0 ponto), totalizando um escore que varia de 0 a 4. 11

### Avaliação da legibilidade das respostas

A legibilidade textual foi avaliada com o programa ALT, ferramenta desenvolvida e validada para o português brasileiro. 8 O programa aplica fórmulas clássicas adaptadas, como Flesch Reading Ease, Gunning Fog, Automated Readability Index (ARI) e Coleman–Liau, e estima a escolaridade mínima necessária para a compreensão do texto. 8

### Análise estatística

As análises estatísticas foram realizadas no programa Jeffreys's Amazing Statistics Program (JASP, gratuito e de código livre), versão 0.19.3.0. As variáveis contínuas foram descritas como valores de média e desvio padrão. Os escores do DISCERN e os índices de legibilidade dos modelos foram comparados pelo teste de Wilcoxon para amostras pareadas, com estimativa do tamanho de efeito pela correlação bisserial de postos ( *rank-biserial correlation* , rrb, em inglês; IC95%). A legibilidade entre os tipos de pergunta foi comparada por análise de variância ( *analusis of vatiance* , ANOVA, em inglês) para medidas repetidas. Diferenças entre as categorias de pergunta para os escores do DISCERN foram analisadas pelo teste de Friedman, seguido do teste *post hoc* de Conover com correções de Bonferroni e de Holm. A confiabilidade interavaliador foi avaliada pelo coeficiente de correlação intraclasse (CCI, modelo de efeitos mistos, tipo 1,1). O nível de significância adotado foi *p* < 0,05.

## Resultados

### Confiabilidade da avaliação e confiabilidade editorial

A confiabilidade interavaliador na aplicação do instrumento DISCERN foi considerada excelente, com CCI1,1 de 0,975 (IC95%: 0,859–0,996), o que demonstra elevada consistência entre os dois revisores independentes.

Quanto à confiabilidade editorial, todas as 48 respostas analisadas receberam pontuação JAMA igual a zero, o que indica ausência de autoria identificável, referências bibliográficas, declaração de conflitos de interesse e data de atualização.

### Comparação geral entre os modelos

O ChatGPT 4.1 mini obteve escore médio DISCERN de 58,7 ± 4,0, e o Gemini 2.5 Flash, 56,3 ± 3,5. Embora a diferença não tenha alcançado significância estatística (W = 6,0; *p* = 0,174), o tamanho do efeito foi máximo (rrb = 1,0; IC95%: 0,554–1,000), indica uma tendência consistente de melhor desempenho do ChatGPT. Esses resultados estão detalhados na Tabela 2 .

**Tabela 2:** Comparação estatística entre os modelos quanto à qualidade informacional e legibilidade

Variável	Teste estatístico	W	Valor de *p*	rrb	IC95% do rrb	Significância
**Escores DISCERN**	Wilcoxon para amostras pareadas	6,0	0,174	1,000	0,554–1,000	Não significativa ( *p* > 0,05)
**Legibilidade (ALT)**	Wilcoxon para amostras pareadas	3,0	1,000	0,000	-0,840 a 0,840	Não significativa ( *p* > 0,05)

Abreviaturas: ALT, Análise de Legibilidade Textual; rrb, *rank-biserial correlation* (“correlação bisserial de postos”).

Quanto à legibilidade, ambos os modelos exigiram em média 13,3 anos de escolaridade para a compreensão dos textos gerados. Não houve diferença significativa entre os modelos nesse aspecto (W = 3,0; *p* = 1,000), com rrb nula (rrb = 0,0; IC95%: -0,840 a 0,840).

**Fig. 1:**
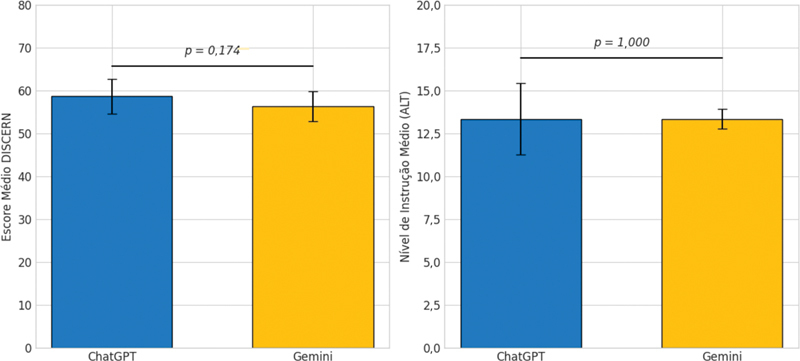
Comparação entre o ChatGPT 4.1 mini e o Gemini 2.5 Flash. ( **A** ) Escores médios de qualidade informacional, avaliados pelo instrumento DISCERN, para cada modelo. ( **B** ) Nível médio de escolaridade necessário para a compreensão das respostas, com base no índice de legibilidade do programa Análise de Legibilidade Textual (ALT). Não houve diferença significativa entre os modelos para os dois parâmetros ( *p* > 0,05).

A Fig. 1 apresenta os dados de forma comparativa. No gráfico A, observam-se os escores médios de qualidade informacional (DISCERN) para cada modelo; no gráfico B, o nível médio de instrução necessário segundo o índice ALT. Em nenhuma das duas análises as diferenças entre os modelos atingiram significância estatística.

### Avaliação estratificada por categoria de pergunta

A análise estatística estratificada conforme a classificação de Rothwell 9 não identificou diferenças significativas nos escores DISCERN entre os grupos (teste de Friedman, Qui quadrado [χ ^2^ ] = 3,71; *p* = 0,156). A comparação dos níveis de legibilidade, mensurados pelo programa ALT, também não revelou diferença estatisticamente significativa entre as categorias (ANOVA, F = 2,11; *p* = 0,321), conforme apresentado na Tabela 3 .

**Tabela 3:** Comparação estatística entre os tipos de pergunta segundo a classificação de Rothwell 9

Variável	Teste	Valor	Valor de *p*	Correção	Significância
**DISCERN** **(geral)**	Friedman	χ ^2^ = 3,71	0,156	–	Não significativa ( *p* > 0,05)
***Post hoc*** **Fato × Política**	Conover	T = 3,54	0,038	Bonferroni = 0,115	Não significativa após ajuste
***Post hoc*** **Fato × Valor**	Conover	T = 4,95	0,038	Bonferroni = 0,115	Não significativa após ajuste
***Post hoc*** **Política × Valor**	Conover	T = 1,41	0,293	Bonferroni = 0,879	Não significativa
**Legibilidade (ALT)**	ANOVA	F = 2,11	0,321	–	Não significativa ( *p* > 0,05)

**Abreviaturas:** χ ^2^ , Qui quadrado; ALT, Análise de Legibilidade Textual; ANOVA, Análise de variância.

Apesar da ausência de significância estatística, observou-se uma tendência descritiva relevante: perguntas de valor obtiveram os maiores escores médios de qualidade (ChatGPT: 61; Gemini: 60), ao passo que as perguntas de fato apresentaram os menores valores (ChatGPT: 54; Gemini: 53). Quanto à legibilidade, as perguntas de fato exigiram menor nível de instrução (∼ 11–12 anos), ao passo que as perguntas de política foram as mais complexas, e alcançaram até 15 anos de escolaridade no caso das respostas do ChatGPT.

**Fig. 2:**
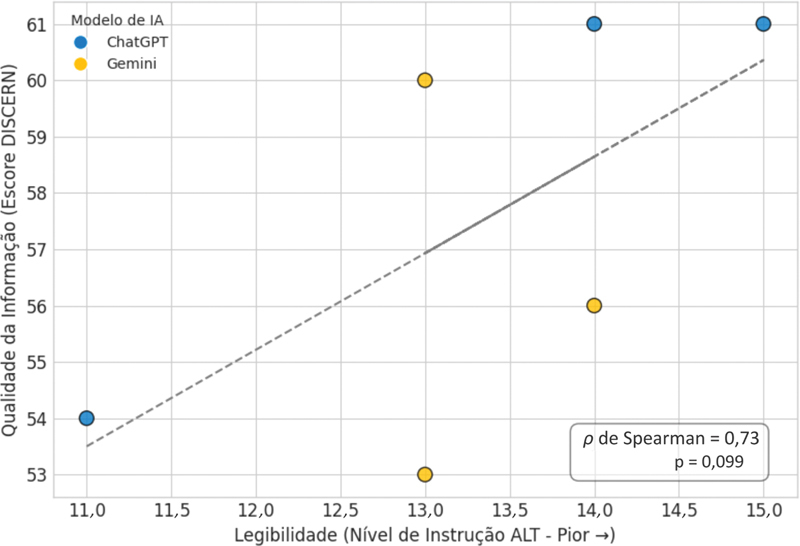
Correlação entre os escores de qualidade informacional (DISCERN) e os índices de legibilidade (ALT) das respostas geradas pelo ChatGPT 4.1 mini e o Gemini 2.5 Flash. A análise demonstrou uma correlação positiva moderada (ρ = 0,73), embora sem significância estatística ( *p* = 0,099). A relação entre qualidade e legibilidade foi observada, mas não foi estatisticamente significativa devido ao tamanho amostral limitado.

A análise de correlação entre qualidade e legibilidade demonstrou associação positiva moderada (ρ = 0,73; *p* = 0,099), sem significância estatística, possivelmente devido ao tamanho amostral limitado ( Fig. 2 ). Já a análise *post hoc* revelou diferença marginal entre os grupos “fato” e “política” ( *p* = 0,038), mas essa diferença perdeu significância após a correção de Bonferroni ( *p* = 0,115).

**Fig. 3:**
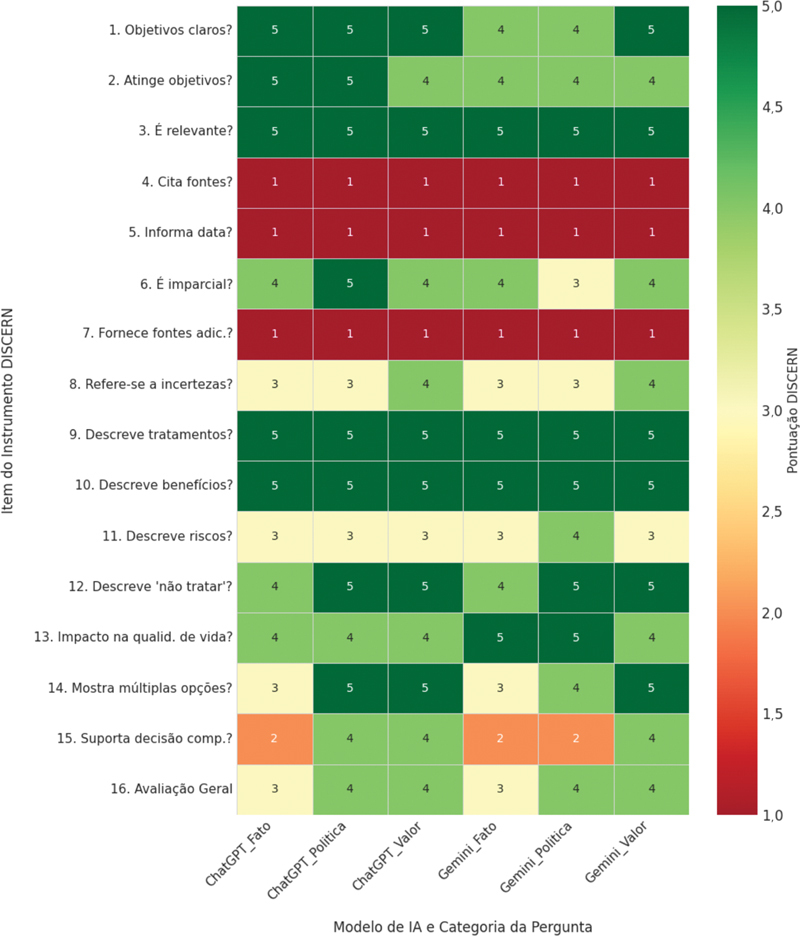
Mapa de calor dos escores médios do DISCERN por modelo de inteligência artificial (IA) e por categoria de pergunta (fato, política e valor). As diferentes intensidades de cor indicam as variações nos escores de qualidade, destacando os pontos fortes e fracos de cada modelo em relação aos tipos de perguntas.

Para uma representação visual detalhada do desempenho dos modelos por categoria de pergunta, a Fig. 3 apresenta um mapa de calor dos escores médios obtidos nos 16 itens do instrumento DISCERN, destacando os pontos fortes e fracos de cada IA em relação aos diferentes tipos de pergunta.

## Discussão

Este estudo identificou um paradoxo no uso de modelos de linguagem na educação em saúde: a produção de conteúdo estruturado e coerente, mas com falhas críticas de confiabilidade editorial e acessibilidade. ChatGPT 4.1 mini e Gemini 2.5 Flash apresentaram escores DISCERN compatíveis com boa qualidade informacional, mas não atenderam a nenhum critério editorial da JAMA e exigiram nível educacional superior ao recomendado para o público leigo, o que limita sua aplicabilidade prática.

Foi observada uma tendência não significativa de escores mais altos no ChatGPT, mas essa diferença deve ser interpretada com cautela, devido ao número reduzido (n = 8) de perguntas por categoria. O delineamento metodológico, inspirado em um estudo prévio escrito em língua inglesa 3 e definido por conveniência, permitiu a comparabilidade, mas reduziu o poder estatístico e aumentou a probabilidade de erro do tipo II, o que pode ter mascarado diferenças reais entre os modelos.

Os achados são compatíveis com os de Warren et al., 3 , que avaliaram informações sobre cirurgia de reparo do manguito rotador em inglês. Nesse estudo, os escores DISCERN situaram-se entre 51 e 55, classificados como “bons”, mas nenhuma resposta atendeu a critérios editoriais da JAMA (autoria, data e referências ausentes). A legibilidade, estimada pelo Flesch–Kincaid Grade Level (FKGL), indicou necessidade de escolaridade entre a 10ª e a 12ª séries do Ensino Médio americano, acima do recomendado pelo National Institutes of Health e pela American Medical Association. Assim como neste trabalho, os autores 3 concluíram que, apesar da coerência textual, o conteúdo gerado por IA falha em rastreabilidade e acessibilidade linguística, o que limita seu valor como material de educação em saúde para pacientes.

De forma semelhante, constatamos ausência de autoria, data e referências, mas esse achado deve ser interpretado à luz do uso intencional de *prompts* simplificados, para simular dúvidas de pacientes. Portanto, os resultados refletem esse cenário específico, e não devem ser generalizados para contextos em que *prompts* médicos estruturados sejam empregados.

Gupta et al. 13 ampliaram essa discussão ao avaliar respostas do ChatGPT 3.5 sobre cirurgia do tendão distal do bíceps. Os escores DISCERN variaram de 59 a 61, novamente classificados como “bons”, mas, tal como neste estudo, os critérios editoriais da JAMA não foram atendidos (JAMA = 0). Além disso, a legibilidade, medida pelo FKGL, aproximou-se de 15, o que sugere necessidade de nível universitário para a compreensão. Esses achados reforçam o padrão observado de que os modelos de linguagem geram conteúdo informacionalmente sólido, mas inacessível ao público leigo e sem rastreabilidade.

Giammanco et al. 4 analisaram seis modelos generativos, Perplexity, Copilot, ChatGPT (versões gratuita e paga) e Gemini (versões gratuita e paga), em perguntas sobre fraturas de clavícula, utilizando diferentes métricas de qualidade (DISCERN, JAMA, Global Quality Score [GQS], EQIP [Ensuring Quality Information for Patients] e legibilidade). Os autores 4 observaram melhor desempenho do Perplexity e do Copilot, cujas respostas foram consideradas mais confiáveis e acessíveis. Em contrapartida, Gemini 1.5 Pro e ChatGPT gratuito obtiveram os piores resultados. É importante destacar que, em nenhum modelo, a legibilidade ficou abaixo da 10ª série do Ensino Médio americano, o que corrobora nossos achados de que a complexidade textual permanece uma barreira para pacientes.

As causas desse padrão parecem estar ligadas à natureza dos LLMs. A elevada qualidade textual pode ser atribuída ao treinamento com literatura científica e médica, que confere aos modelos a capacidade de replicar a estrutura formal e a lógica argumentativa de respostas clínicas bem elaboradas. 14 Por outro lado, a ausência de critérios editoriais da JAMA reflete o fato de que tais modelos não foram projetados para atribuição de autoria ou referência de fontes, pois seu objetivo primário é a geração fluente de linguagem, não a rastreabilidade informacional. 15 Adicionalmente, a elevada complexidade linguística parece decorrer do registro estilístico predominante nesses modelos, derivado de seu *corpus* de treinamento (conjunto massivo de textos utilizados para treinar o modelo), sendo pouco responsivo à simplificação textual na ausência de instruções explícitas. 16

### Implicações clínicas e recomendações

Modelos de linguagem produzem respostas coerentes que podem induzir pacientes a superestimar sua validade científica, apesar da ausência de rastreabilidade e critérios editoriais. Esse cenário evidencia a necessidade de que profissionais de saúde realizem avaliação crítica das informações digitais, integrando análise da validade científica, contextualização clínica e comunicação clara e segura, de forma a apoiar decisões baseadas em evidências.

### Limitações

O número reduzido de perguntas comprometeu o poder estatístico e restringe a generalização dos achados. A análise foi pontual, sem avaliação da consistência temporal das respostas, e o desempenho pode variar em versões futuras dos modelos. Além disso, não houve avaliação direta por pacientes, o que impede inferências sobre compreensão prática e utilidade percebida.

### Perspectivas futuras

Pesquisas futuras devem se centrar em estratégias que aumentem a segurança e a aplicabilidade clínica da IA em saúde utilizando os modelos atualmente disponíveis. Entre as prioridades estão: 1) estudos longitudinais que avaliem o impacto das respostas de IA sobre decisões clínicas, adesão terapêutica e autogestão de condições crônicas; 2) validação de estratégias de *prompt engineering* , com instruções estruturadas que orientem os modelos a gerar informações mais precisas, compreensíveis e confiáveis; e 3) análise de abordagens que combine respostas de IA com orientação profissional, identificando formas eficazes de incorporar essas ferramentas à prática clínica.

## Conclusão

O presente estudo mostrou que, em português brasileiro, os modelos ChatGPT 4.1 mini e Gemini 2.5 Flash geraram respostas sobre cirurgia de reparo do manguito rotador com qualidade informacional considerada “boa” pelo DISCERN. No entanto, ambos apresentaram pontuação nula nos critérios da JAMA e legibilidade acima do nível recomendado para o público leigo, o que limita a sua aplicabilidade como ferramenta de educação em saúde para pacientes.
